# Potential Clinical Value of 5-Hydroxytryptamine Receptor 3C as a Prognostic Biomarker for Lung Cancer

**DOI:** 10.1155/2021/1901191

**Published:** 2021-11-15

**Authors:** Jiun-Rung Chen, Ming-Shyan Huang, Yi-Chen Lee, Min-His Lin, Yi-Fang Yang

**Affiliations:** ^1^Division of Pulmonary & Respiratory Medicine, Department of Internal Medicine, E-Da Cancer Hospital, Kaohsiung, Taiwan; ^2^Department of Internal Medicine E-Da Cancer Hospital School of Medicine, I-Shou University, Kaohsiung, Taiwan; ^3^Department of Anatomy, School of Medicine, College of Medicine, Kaohsiung Medical University, Kaohsiung, Taiwan; ^4^Division of Chest Medicine, Kaohsiung Veterans General Hospital, Kaohsiung, Taiwan; ^5^Department of Medical Education and Research, Kaohsiung Veterans General Hospital, Kaohsiung, Taiwan

## Abstract

Ion channels and pumps not only regulate membrane potential, ion homeostasis, and electric signaling in excitable cells but also contribute to cell proliferation, migration, apoptosis, and differentiation. Channel proteins and ion pumps can form macromolecular complexes with signaling molecules, including growth factors and cell adhesion molecules. Serotonin (5-hydroxytryptamine (5-HT)) promotes the proliferation of various cancer cell types mediated through the activation of the 5-HT receptor (HTR). Only HTR3 is a ligand-gated ion channel. However, the role of the HTR3 family of HTRs in lung cancer has not been adequately evaluated. We evaluated the relationship between the HTR3 family of HTRs and lung cancer patients' survival using Kaplan–Meier analyses and examined the expression levels of target proteins using immunohistochemistry. In this study, we found that *HTR3C* was amplified with high frequency in lung cancer patients, and HTR3C protein expression levels were significantly associated with lymph node metastasis and distant metastasis in lung cancer tissues. Survival analysis using the log-rank test demonstrated a decrease in disease-free survival (DFS) and overall survival (OS) rates among the high-level HTR3C expression group compared with the low-level HTR3C expression group. We also evaluated the risk factors associated with lung cancer. The univariate and multivariate analyses of DFS and OS showed that HTR3C expression was a significant predictor of patient outcomes. Taken together, these data demonstrated that HTR3C expression levels were associated with poor DFS and OS in lung cancer patients, indicating that HTR3C can serve as a useful predictive biomarker for lung cancer.

## 1. Introduction

Lung cancer is a malignant tumor type and is currently the leading cause of cancer-related deaths worldwide. As high as 80% of lung cancer cases are categorized as non-small-cell lung cancer (NSCLC) [1, 2]. Approximately 70% of newly diagnosed lung cancer patients require systemic treatment due to locally advanced or metastatic disease. However, lung cancer patients often have poor prognoses, low 5-year survival rates, and high mortality rates, most likely due to the high metastatic rate of lung cancer [2–4]. According to previous studies, ion channels contribute to the malignant behaviors of cancer cells, including migration, invasion, cell cycle control, and metastasis [5]. Ion channel upregulation and increased activity have been reported in response to mitogen exposure [6–8], and accumulating evidence supports the existence of a direct link between transmembrane ion flow and carcinogenesis [9, 10].

Serotonin (5-hydroxytryptamine (5-HT)) stimulates cancer cell growth *in vitro* [11], mediated by the activation of 5-HT receptors (HTRs, 5-HT_1_ to 5-HT_7_). HTRs can be categorized as G protein-coupled receptors (GPCRs) and ligand-gated ion channels. Among known HTRs, only 5-HT_3_ receptor has been identified as a ligand-gated ion channel, whereas other identified HTRs are GPCRs activated by cAMP (an intracellular messenger) [12]. The 5-HT_3_ receptor family includes HTR3A, HTR3B, HTR3C, HTR3D, and HTR3E [13]. HTR3A and HTR3B assemble into 5-HT_3_ receptors, but HTR3C, HTR3D, and HTR3E provide molecular diversity within the family [14].

In this study, we investigated whether the 5-HT_3_ ionotropic receptor family was associated with a malignant lung cancer phenotype. We examined the genetic variations in members of the HTR3 family in NSCLC patient data contained in a dataset obtained from The Cancer Genome Atlas (TCGA) and evaluated the relationship between HTR3 family mutations and patient survival using a Kaplan–Meier analysis. Based on bioinformatics analysis, we identified targets induced by HTR3 activation and examined relationships between these protein expression levels and clinical outcomes in patients with lung cancer.

## 2. Materials and Methods

### 2.1. In Silico Genetic Profiles and Kaplan–Meier Analysis of HTR3 Family Members

Genetic variations in HTR3 family members and mutation frequency were analyzed using the online TCGA dataset (cBioPortal, https://www.cbioportal.org/). Kaplan–Meier analysis (overall survival (OS)) was examined using a lung cancer microarray dataset [15].

### 2.2. Specimens

Lung cancer patients were recruited from E-Da Hospital after obtaining IRB approval (EMRP-107-069). All specimens were subjected to immunohistochemical staining to evaluate the expression of HTR3C. Histological grade was classified according to the recommendations of the World Health Organization (WHO). Histological diagnostic features, including tumor size, local invasion, lymph node involvement, distant metastasis, and final disease stage, were determined according to the American Joint Committee on Cancer (AJCC) tumor node metastasis (TNM) classification system for lung cancer [2]. Follow-up lasted up to 200 months.

### 2.3. Immunohistochemistry

Immunohistochemistry (IHC) was performed according to the manufacturer's instructions. In brief, tissue slides were deparaffinized, rehydrated with a graded ethanol solution, and then rinsed with distilled water. Antigen retrieval was performed by immersing the slides in retrieval solution (pH6.0) for 15 min at 96°C. The tissue slides were incubated with primary antibody against HTR3C (1 : 50; #ab199148; Abcam, Cambridge, United Kingdom) for 30 min at room temperature. The antigen-antibody complexes were detected by the avidin-biotin-peroxidase method (using 3,3′-diaminobenzidine as a chromogenic substrate). A negative control incubated immunoglobulin fraction of nonimmune rabbit serum for each staining. HTR3C expression was classified according to the H-score, which was calculated as the percentage of positively stained cells multiplied by the staining intensity.

### 2.4. Statistical Analysis

All statistical analyses were performed using SPSS 19.0 statistical package. Associations between HTR3 or downstream targets and tumor stage, tumor grade, age at diagnosis, tumor size, lymph nodes status, and recurrence were analyzed using the two-sided *χ*^2^ test. Survival curves were generated using Kaplan–Meier estimates, and significant differences between curves were evaluated using the two-sided log-rank test. Hazard ratios (HR) and 95% confidence intervals (CIs) were computed for univariate and multivariate Cox regression models to investigate the correlation between clinicopathological characteristics and survival. *P* < 0.05 was considered significant.

## 3. Results

### 3.1. In Silico Genetic Profiles of HTR3 Family Members in NSCLC

**Figure 1:**
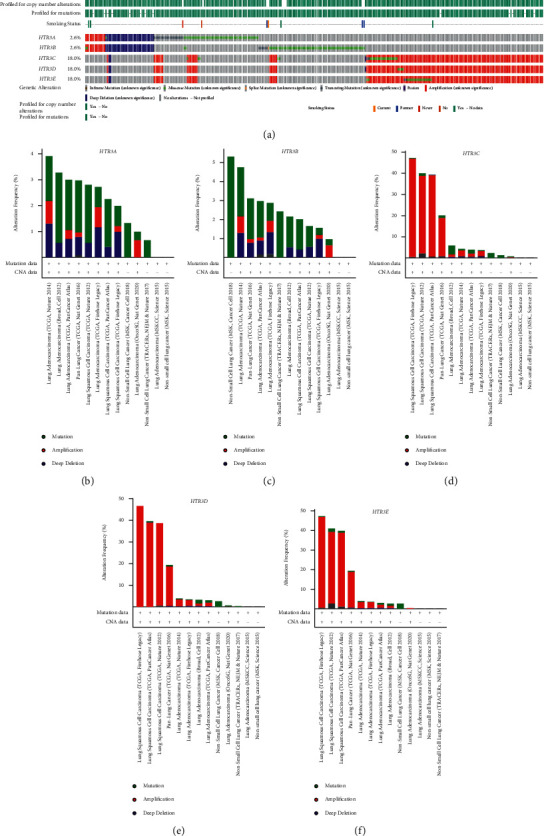
*HTR3C*, *HTR3D*, and *HTR3E* showed the highest amplification levels in NSCLC. (a) OncoPrint showing *HTR3C*, *HTR3D*, and *HTR3E* amplification in 18% of NSCLC cases. Colors indicate the type of genetic mutation (missense, in-frame, truncated, amplification, deletion, and fusion), and different cohorts are listed below the OncoPrint. Cancer type summary of *HTR3A* (b), *HTR3B* (c), *HTR3C* (d), *HTR3D* (e), and *HTR3E* (f) according to the lung cancer cohorts (cBioPortal). CAN = copy number alterations.

First, we evaluated the genetic profiles of *HTR3* family members (HTR3A–HTR3E) in NSCLC patients using a publicly available dataset (cBioPortal). The genetic profiles of HTR3 family members were obtained from 6,122 NSCLC samples, which revealed that *HTR3C*, *HTR3D,* and *HTR3E* were significantly amplified in NSCLC genetic profiles (Figure 1). We next analyzed the frequency of gene amplification co-occurrence within the same samples from lung cancer patients, which showed high frequencies of co-occurrence for some gene amplification pairs within the same patient, including *HTR3C*/*HTR3D*, *HTR3C*/*HTR3E*, and *HTR3D*/*HTR3E*, whereas the co-occurrence of gene amplification was not observed for other gene pairs (*HTR3A*/*HTR3B*, *HTR3B*/*HTR3D*, and *HTR3B*/*HTR3C*) (Supplementary Table 1).

### 3.2. High HTR3C Expression Levels in Tumor Tissues Are Associated with Poor Clinical Outcomes in Patients with NSCLC

**Figure 2:**
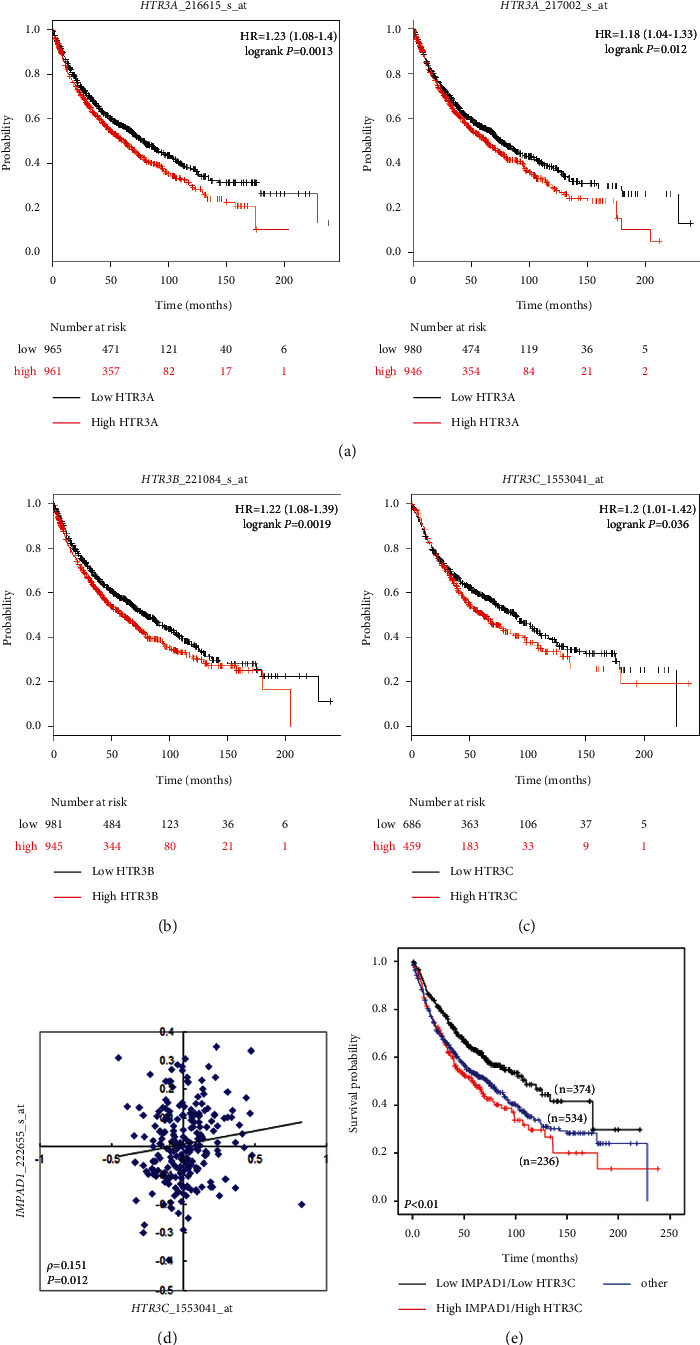
HTR3 family as a potential target for the prevention of lung cancer progression. Kaplan–Meier curve for overall survival, according to the mRNA expression *HTR3A* (a), *HTR3B* (b), and *HTR3C* (c), using publicly available lung cancer microarray datasets. HR = hazard ratio. (d) Analysis correlation of *HTR3C* and *IMPAD1* mRNA expression by using microarray datasets of lung cancer (NCBI/GEO/GSE31210). (e) Relative mRNA expression levels of *HTR3C* and *IMPAD1* in lung cancer patients (online KM plotter).

To evaluate which members of the HTR3 family were associated with lung cancer progression, we used gene expression microarray analysis, followed by Kaplan–Meier survival curve analysis, which showed that *HTR3A*, *HTR3B*, and *HTR3C* expression levels were associated with poor OS (Figures 2(a)–2(c)). The results of the survival analysis suggested that the HTR3 family plays important roles in lung cancer progression. We evaluated the direct interaction partners of HTR3C using the BioGRID website and found that inositol monophosphatase domain-containing 1 (IMPAD1) interacts with HTR3C (Supplementary Table 2). IMPAD1 acts as a mitochondrial electron transport inhibitor and promotes lung cancer metastasis [16]. We further examined the correlation between *HTR3C* and *IMPAD1* mRNA levels, which showed that *HTR3C* mRNA levels were positively correlated with *IMPAD1* mRNA levels in lung cancer tissues (GSE31210; Figure 2(d)). The combination of *HTR3C* and *IMPAD1* expression was further evaluated by comparing lung cancer patients with high *HTR3C* and high *IMPAD1* expression against patients with low *HTR3C* and low *IMPAD1* expression using the Kaplan–Meier plotter website [17]. The high *HTR3C/*high *IMPAD1* expression group was associated with shorter OS than the low *HTR3C*/low *IMPAD1* expression group among lung cancer patients (Figure 2(e)). Moreover, *HTR3C* gene amplification correlated with *HTR3C* mRNA expression in lung cancer patients (Supplementary Figure 1). Therefore, we further examined the characteristics of HTR3C and its contributions to lung cancer.

### 3.3. HTR3C Protein Upregulation Is Associated with Poor Clinical Outcomes in Lung Cancer

**Figure 3:**
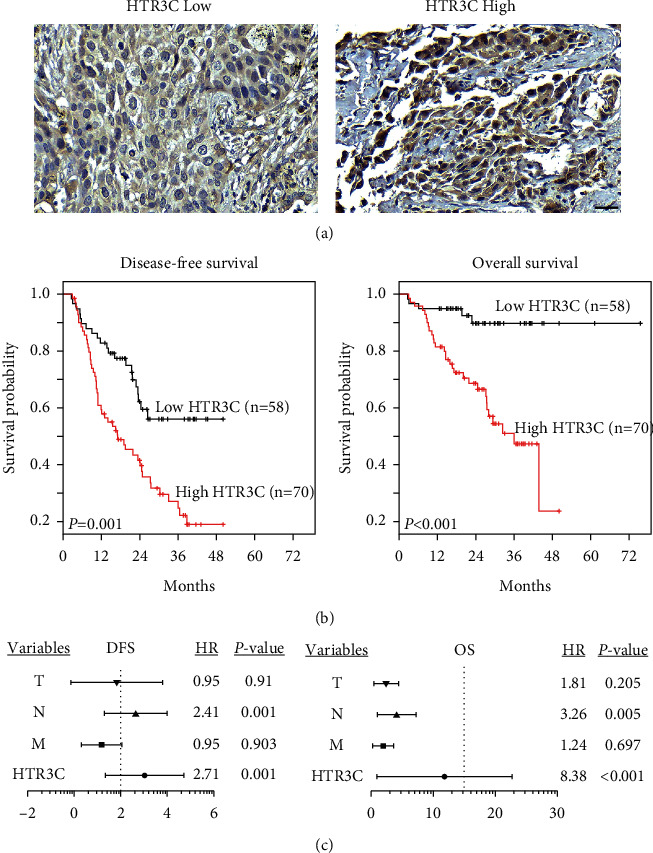
HTR3C is overexpressed in lung cancer and correlates with worse survival. (a) Representative images of HTR3C IHC staining in lung cancer tissues. (b) Kaplan–Meier curve for disease-free survival (DFS) and overall survival (OS) among lung cancer patients according to HTR3C expression. (c) Hazard ratios were determined by multivariate Cox regression analysis.

To evaluate HTR3C protein levels in lung cancer, we examined the expression of HTR3C in primary lung tumors from 128 patients using IHC and correlated HTR3C expression with the clinicopathological characteristics of patients. As shown in Figure 3(a), HTR3C protein expression in cancer tissues was classified into low and high expression level groups. We found that high HTR3C expression levels in lung cancer tissues were significantly associated with positive lymph node metastasis (N status; *P*=0.028), positive distant metastasis (M status; *P*=0.042), recurrence (*P* < 0.001), and cancer death (*P* < 0.001; Table 1). Survival analysis using the log-rank test demonstrated decreased disease-free survival (DFS) and OS rates for the high HTR3C expression level group compared with those for the low HTR3C expression level group (*P* < 0.01; Figure 3(b)). We also evaluated the risk factors associated with lung cancer. Univariate and multivariable Cox regression analyses were used to estimate HRs, which showed that HTR3C expression level was a significant predictor of both OS and DFS (Figure 3(c), Tables 2 and 3).

### 3.4. HTR3C Expression Positively Correlates with TTN in Lung Cancer Patients

**Figure 4:**
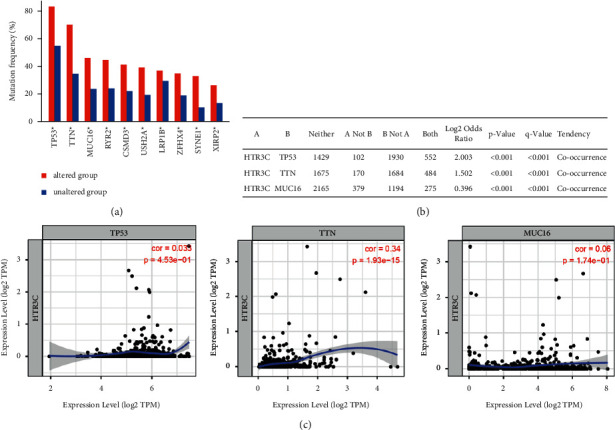
Correlation analysis between *HTR3C* and oncogene expression in lung cancer patients. (a) Frequency of genomic alterations in tumors obtained from lung cancer patients. ^*∗*^*P* < 0.05. (b) Table of major co-occurring genomic alterations in *HTR3C-*amplified NSCLC. (c) Analysis of the correlations between *HTR3C* and *TP53*/*TTN*/*MUC16* mRNA expression levels using TIMER.

To evaluate whether the genetic alteration events reflect the HTR3C expression level in lung cancer patients, we screened and listed the most commonly mutated gene in lung cancer patients. Our results indicated that these genes' (*TP53*, *TTN*, *MUC16*, *RYR2*, *CSMD3*, *USH2A*, *LRP1B*, *ZFHX4*, *SYNE1*, and *XIRP2*) mutations are frequently present in clinical lung cancer patients (Figure 4(a)). We focus on top three genes; Figure 4(b) shows the co-occurrence between *HTR3C* gene alterations and a series of core lung cancer-associated genes, including *TP53*, *TTN*, and *MUC16*. We further examined the correlation between the mRNA expression levels of *HTR3C* and those of *TP53*, *TTN*, and *MUC16* using TIMER [18]. The results showed that *HTR3C* mRNA levels were positively correlated with *TTN* mRNA levels in lung cancer tissues (Figure 4(c)).

## 4. Discussion

In this study, HTR3 family members were assessed in NSCLC patients, which showed that *HTR3C*, *HTR3D*, and *HTR3E* were amplified with high frequency among lung cancer patients. *HTR3A*, *HTR3B*, and *HTR3C* mRNA expression levels were associated with poor OS. Based on the IHC results, high protein expression levels of HTR3C were associated with lymph node metastasis, distant metastasis, and recurrence in lung cancer patients. Moreover, lung cancer patients presenting with high HTR3C protein levels were associated with shorter OS and DFS than patients with low HTR3C protein levels. *TP53*, *TNT*, and *MUC16* mutations occurred at high frequencies among NSCLC patients and co-occurrence with altered *HTR3C*. *HTR3C* mRNA levels were positively correlated with *TNT* mRNA levels in lung cancer tissues. These findings suggest that HTR3C may serve as a prognostic marker in lung cancer.

5-HT can affect tumor behaviors via the activation of HTRs. *In vitro* studies have shown that 5-HT promotes cell growth in cancer cell lines, including small-cell lung cancer (SCLC) cells, mediated by the activation of the HTRs 5-HT_1A_ and 5-HT_1D_ [11, 19]. The proliferation of endothelial cells is stimulated by the activation of the HTRs 5-HT_1_, 5-HT_2_, and 5-HT_3_, and 5-HT_2_ activation was shown to mediate similar effects in aortic smooth muscle cells [20]. 5-HT activates mitogen-activated protein kinase (MAPK) and the phosphoinositide 3-kinase (PI3K)/protein kinase B (AKT) signaling pathway to promote malignant behaviors in prostate cancer cells [21] through several receptor subtypes, promoting disease progression in prostate cancer patients [11, 22]. Wang et al. showed that the HTRs 5-HT_3_ and 5-HT_4_, which are located on the mitochondria membrane, modulate the respiration control ratio (RCR) in cardiac mitochondria. Moreover, HTR3 has been reported to regulate mitochondrial functions, altering ATP contents and lactate dehydrogenase (LDH) release [23]. Our data showed that HTR3C expression was significantly associated with lymph node metastasis, distant metastasis, and recurrence in lung cancer patients. In the BioGRID dataset, HTR3C interaction with the oncogene IMPAD1 was shown to promote lung cancer metastasis via the inhibition of mitochondrial electron transport [16]. The high *HTR3C*/high *IMPAD1* expression cohort showed a significant decrease in OS compared with the low *HTR3C*/low *IMPAD1* expression cohort (Figure 2(e)). These findings suggested that the HTR3C/IMPAD1 axis may play a role in lung cancer progression.

Previously, programmed cell death ligand (PD-L1) expression as detected by IHC was designated as the first Food and Drug Administration (FDA) approved biomarker for the monitoring of patient's responsiveness to immune checkpoint blockade (ICB) immunotherapy. In addition, several studies have shown that tumor mutational burden (TMB) can serve as a biomarker for predicting the responsiveness to ICB-based immunotherapy for a variety of tumor types [24, 25]. The DNA damage response (DDR) pathway [26], deficient mismatch repair (dMMR) [27], *TP53*/*KRAS* comutation [28], and *TTN* (Titin) mutations [29] have also been established as predictive biomarkers for ICB treatment. Our study revealed that *TP53*, *TNT*, and *MUC16* mutations were co-occurrence with altered *HTR3C* in NSCLC patients. Furthermore, *HTR3C* mRNA expression levels were correlated with *TNT* mRNA expression levels in NSCLC patients. We could not exclude the possibility that *HTR3C* amplification or expression may also be correlated with the response rate to ICB; however, this is the first study to evaluate alterations in the *HTR3C* gene and protein expression in lung cancer patients.

This is the first study to evaluate genetic variations in the *HTR3* family, which revealed *HTR3C*, *HTR3D*, and *HTR3E* gene amplification in NSCLC patients. High levels of HTR3C protein expression were correlated with shorter OS and DFS compared with low levels of HTR3C protein expression, and Cox regression analyses demonstrated that HTR3C expression levels could serve as an independent prognostic factor for lung cancer outcomes.

## Figures and Tables

**Table 1:** Correlation between HTR3C expression levels and various clinicopathological characteristics in lung cancer.

Variables	Item	Patient no. (%)	HTR3C				*P* value
			Low (≤92.62)		High (>92.62)		
			No.	%	No.	%	
		128 (100.0)	58	45.3	70	54.7	
Age (y)	≤70	98 (76.6)	41	70.7	57	81.4	0.153^a^
	>70	30 (23.4)	17	29.3	13	18.6	

Sex	Female	46 (35.9)	18	31.0	28	40.0	0.293^a^
	Male	82 (64.1)	40	69.0	42	60.0	

Grade	I	29 (22.7)	10	17.2	19	27.1	0.183^a^
	II/III	99 (77.3)	48	82.8	51	72.9	

T status	T1/T2	107 (83.6)	45	77.6	62	88.6	0.095^a^
	T3/T4	21 (16.4)	13	22.4	8	11.4	

N status	Negative (N0)	68 (53.1)	37	63.8	31	44.3	0.028^a^
	Positive (N1/N2/N3)	60 (46.9)	21	36.2	39	55.7	

M status	Negative	110 (85.9)	54	90.2	56	76.6	0.042^b^
	Positive	18 (14.1)	4	9.8	14	23.4	

Histology	Adenocarcinoma	97 (78.2)	43	75.4	54	80.6	0.488^a^
	Squamous cell carcinoma	27 (21.8)	14	24.6	13	19.4	

Performance	0	85 (66.4)	40	69.0	45	64.3	0.577^a^
	1	43 (33.6)	18	31.0	25	35.7	

Smoking status	Never	76 (59.4)	34	58.6	42	60.0	0.472^a^
	Former	22 (17.2)	8	13.8	14	20.0	
	Current	30 (23.4)	16	27.6	14	20.0	

Recurrence	No	58 (45.3)	37	63.8	21	30.0	<0.001^a^
	Yes	70 (54.7)	21	36.2	49	70.0	

Cancer death	No	93 (72.7)	53	91.4	40	57.1	<0.001^b^
	Yes	35 (27.3)	5	8.6	30	42.9	

^a^ *P* value was calculated by the chi-square test. ^b^*P* value was calculated by Fisher's exact test.

**Table 2:** Univariate and multivariate analysis of overall survival for lung cancer.

Variables	Item	Univariate			Multivariate		
		HR	95% CI	*P* value	HR	95% CI	*P* value
Age (y)	>70	1.62	(0.79, 3.30)	0.188	2.37	(0.98, 5.73)	0.056
	≤70	1.00			1.00		

Sex	Male	1.50	(0.70, 3.21)	0.295	0.74	(0.26, 2.11)	0.576
	Female	1.00			1.00		

Grade	II/III	1.82	(0.70, 4.68)	0.218	1.05	(0.33, 3.33)	0.928
	I	1.00			1.00		

T status	T4/T3	1.44	(0.65, 3.18)	0.364	1.81	(0.72, 4.52)	0.205
	T2/T1	1.00			1.00		

N status	Positive	3.43	(1.64, 7.19)	0.001	3.26	(1.42, 7.48)	0.005
	Negative	1.00			1.00		

M status	Positive	3.35	(1.59, 7.08)	0.002	1.24	(0.42, 3.73)	0.697
	Negative	1.00			1.00		

Histology	Squamous cell carcinoma	2.72	(1.36, 5.44)	0.005	3.98	(1.59, 9.94)	0.003
	Adenocarcinoma	1.00			1.00		

Performance	1	4.55	(2.28, 9.07)	<0.001	5.34	(2.24, 12.73)	<0.001
	0	1.00			1.00		

Smoking status	Current	2.54	(1.19, 5.41)	0.016	1.71	(0.56, 5.21)	0.345
	Former	1.89	(0.79, 4.52)	0.152	2.09	(0.70, 6.26)	0.187
	Never	1.00			1.00		

HTR3C	High	5.69	(2.20, 14.73)	<0.001	8.38	(2.93, 23.98)	<0.001
	Low	1.00			1.00		

HR = hazard ratio; CI = confidence interval.

**Table 3:** Univariate and multivariate analysis of disease-free survival for lung cancer.

Variables	Item	Univariate			Multivariate		
		HR	95% CI	*P* value	HR	95% CI	*P* value
Age (y)	>70	1.22	(0.71, 2.12)	0.471	1.29	(0.68, 2.42)	0.438
	≤70	1.00			1.00		

Sex	Male	0.90	(0.55, 1.47)	0.680	0.88	(0.48, 1.61)	0.677
	Female	1.00			1.00		

Grade	II/III	2.04	(0.52, 1.81)	0.037	1.87	(0.88, 4.00)	0.105
	I	1.00			1.00		

T status	T4/T3	0.97	(0.51, 1.64)	0.919	0.95	(0.45, 2.03)	0.901
	T2/T1	1.00			1.00		

N status	Positive	2.98	(1.81, 4.90)	<0.001	2.41	(1.42, 4.08)	0.001
	Negative	1.00			1.00		

M status	Positive	2.41	(1.37, 4.23)	0.002	0.95	(0.43, 2.13)	0.903
	Negative	1.00			1.00		

Histology	Squamous cell carcinoma	1.73	(1.02, 2.96)	0.043	1.94	(1.03, 3.65)	0.041
	Adenocarcinoma	1.00			1.00		

Performance	1	2.24	(1.40, 3.58)	0.001	2.16	(1.18, 3.94)	0.012
	0	1.00			1.00		

Smoking status	Current	1.06	(0.60, 1.90)	0.835	1.08	(0.49, 2.37)	0.856
	Former	1.07	(0.58, 1.97)	0.840	1.18	(0.57, 2.45)	0.654
	Never	1.00			1.00		

HTR3C	High	2.37	(1.42, 3.96)	0.001	2.71	(1.51, 4.85)	0.001
	Low	1.00			1.00		

HR = hazard ratio; CI = confidence interval.

## Data Availability

The datasets supporting the conclusions of this article are included within the article.

## Supplementary Materials

Supplementary Materials Figure S1: *HTR3C* amplification and expression levels in NSCLC as reported by cBioPortal. Table S1: HTR3 family members' mutual exclusivity. Table S2: displaying 121 total unique interactors of HTR3C.
