# Supplementary material for: Potential Clinical Value of 5-Hydroxytryptamine Receptor 3C as a Prognostic Biomarker for Lung Cancer
Source: J Oncol. 2021 Nov 15;2021:1901191. doi: 10.1155/2021/1901191 (PMC8639264; doi:10.1155/2021/1901191)
Supplement: Supplementary Materials — Figure S1: HTR3C amplification and expression levels in NSCLC as reported by cBioPortal. Table S1: HTR3 family members' mutual exclusivity. Table S2: displaying 121 total unique interactors of HTR3C. [file 1901191.f1.docx]

**Potential clinical value of 5-hydroxytryptamine receptor 3C as a prognostic biomarker for lung cancer**

Jiun-Rung Chen^1†^ **iD**, Ming-Shyan Huang^2†^ **iD**, Yi-Chen Lee^3^ **iD**, Min-His Lin ^4^ and Yi-Fang Yang^5*^ **iD**

https://orcid.org/0000-0003-3211-4312(Jiun-Rung Chen)

https://orcid.org/0000-0001-8180-2213 (Ming-Shyan Huang)

https://orcid.org/0000-0002-4889-095X (Yi-Chen Lee)

https://orcid.org/0000-0001-7425-3156 (Yi-Fang Yang)

^1^ Division of Pulmonary & Respiratory Medicine, Department of Internal Medicine, E-Da Cancer Hospital, Kaohsiung, Taiwan

^2^ Department of Internal Medicine E-DA Cancer Hospital School of Medicine

I-Shou University Kaohsiung, Taiwan

^3^ Department of Anatomy, School of Medicine, College of Medicine, Kaohsiung Medical University, Kaohsiung, Taiwan

^4^ Division of Chest Medicine, Kaohsiung Veterans General Hospital, Kaohsiung, Taiwan

^5^ Department of Medical Education and Research, Kaohsiung Veterans General Hospital, Kaohsiung, Taiwan

†Jiun-Rung Chen and Ming-Shyan Huang contributed equally to this work.

Correspondence to: Yi-Fang Yang, PhD

Department of Medical Education and Research, Kaohsiung Veterans General

Hospital, No. 386, Dajhong 1st Rd., Zuoying Dist., Kaohsiung City 813414, Taiwan. Phone: 886-7-342-2121 # 71592; Fax: 886-7-342-2288; E-mail: yvonne845040@gmail.com

**Running Title**:

HTR3C associated with poor survival in NSCLC.

**Conflict of interest:** The authors declare that they have no conflicts of interest.

Inventory of all Supplemental Information

Supplemental Data

Figure S1 Related to Figure 2

Supplementary Tables

Supplementary Table 1 Related to Figure 1

Supplementary Table 2 Related to Figure 2

**SUPPLEMENT FIGURE1.** ***HTR3C* amplification and expression levels in NSCLC as reported by cBioPortal.**

**SUPPLEMENTARY TABLE S1.HTR3 family members mutual exclusivity**

| **A** | **B** | **Neither** | **A Not B** | **B Not A** | **Both** | **Log2 Odds Ratio** | ***P*-Value** | **q-Value** | **Tendency** |
| --- | --- | --- | --- | --- | --- | --- | --- | --- | --- |
| HTR3C | HTR3D | 3335 | 52 | 24 | 602 | >3 | <0.001 | <0.001 | Co-occurrence |
| HTR3C | HTR3E | 3330 | 53 | 29 | 601 | >3 | <0.001 | <0.001 | Co-occurrence |
| HTR3D | HTR3E | 3359 | 24 | 28 | 602 | >3 | <0.001 | <0.001 | Co-occurrence |
| HTR3A | HTR3B | 3832 | 67 | 76 | 38 | >3 | <0.001 | <0.001 | Co-occurrence |
| HTR3B | HTR3E | 3276 | 107 | 623 | 7 | -1.539 | 0.001 | 0.003 | Mutual exclusivity |
| HTR3B | HTR3D | 3280 | 107 | 619 | 7 | -1.528 | 0.002 | 0.003 | Mutual exclusivity |
| HTR3B | HTR3C | 3254 | 105 | 645 | 9 | -1.209 | 0.006 | 0.009 | Mutual exclusivity |

**SUPPLEMENTARY TABLE S2. Displaying 121 total unique interactors of HTR3C**

| Official Symbol Interactor B | Experimental System | Experimental System Type | Throughput | Score |
| --- | --- | --- | --- | --- |
| MBOAT7 | Affinity Capture-MS | physical | High Throughput | 0.8635 |
| LSR | Affinity Capture-MS | physical | High Throughput | 0.925 |
| METTL25 | Affinity Capture-MS | physical | High Throughput | 0.99992 |
| TMEM87A | Affinity Capture-MS | physical | High Throughput | 0.87668 |
| PIGO | Affinity Capture-MS | physical | High Throughput | 0.99382 |
| MGAT1 | Affinity Capture-MS | physical | High Throughput | 0.86463 |
| ALG9 | Affinity Capture-MS | physical | High Throughput | 0.993 |
| GPAA1 | Affinity Capture-MS | physical | High Throughput | 0.91322 |
| ENTPD6 | Affinity Capture-MS | physical | High Throughput | 0.87118 |
| TMTC3 | Affinity Capture-MS | physical | High Throughput | 0.99994 |
| KIAA2013 | Affinity Capture-MS | physical | High Throughput | 0.75566 |
| NSDHL | Affinity Capture-MS | physical | High Throughput | 0.8752 |
| TPST2 | Affinity Capture-MS | physical | High Throughput | 0.99992 |
| MANEAL | Affinity Capture-MS | physical | High Throughput | 0.89226 |
| SLC38A10 | Affinity Capture-MS | physical | High Throughput | 0.9876 |
| LEMD3 | Affinity Capture-MS | physical | High Throughput | 0.98854 |
| SLC35B2 | Affinity Capture-MS | physical | High Throughput | 0.98655 |
| SPPL2B | Affinity Capture-MS | physical | High Throughput | 0.99956 |
| TMED4 | Affinity Capture-MS | physical | High Throughput | 0.80832 |
| DGCR2 | Affinity Capture-MS | physical | High Throughput | 0.83424 |
| C1GALT1C1 | Affinity Capture-MS | physical | High Throughput | 0.89285 |
| TMEM186 | Affinity Capture-MS | physical | High Throughput | 0.99985 |
| C3orf58 | Affinity Capture-MS | physical | High Throughput | 0.76676 |
| ITPRIP | Affinity Capture-MS | physical | High Throughput | 0.97355 |
| PGAP1 | Affinity Capture-MS | physical | High Throughput | 0.79488 |
| SEC11C | Affinity Capture-MS | physical | High Throughput | 0.99999 |
| ATP11C | Affinity Capture-MS | physical | High Throughput | 0.98378 |
| RDH11 | Affinity Capture-MS | physical | High Throughput | 0.86159 |
| GRAMD1A | Affinity Capture-MS | physical | High Throughput | 0.96512 |
| FZD2 | Affinity Capture-MS | physical | High Throughput | 0.99734 |
| NHLRC3 | Affinity Capture-MS | physical | High Throughput | 0.82955 |
| TMEM30A | Affinity Capture-MS | physical | High Throughput | 0.81813 |
| NETO2 | Affinity Capture-MS | physical | High Throughput | 0.95875 |
| WLS | Affinity Capture-MS | physical | High Throughput | 0.95316 |
| FAM69B | Affinity Capture-MS | physical | High Throughput | 0.93364 |
| ERMP1 | Affinity Capture-MS | physical | High Throughput | 0.99776 |
| POMT2 | Affinity Capture-MS | physical | High Throughput | 0.98247 |
| FUT8 | Affinity Capture-MS | physical | High Throughput | 0.9235 |
| PIGM | Affinity Capture-MS | physical | High Throughput | 0.99807 |
| UGT3A2 | Affinity Capture-MS | physical | High Throughput | 0.99947 |
| IMPAD1 | Affinity Capture-MS | physical | High Throughput | 0.92654 |
| TMPPE | Affinity Capture-MS | physical | High Throughput | 0.97688 |
| FAM69A | Affinity Capture-MS | physical | High Throughput | 0.99887 |
| SEMA4C | Affinity Capture-MS | physical | High Throughput | 0.84172 |
| ABHD3 | Affinity Capture-MS | physical | High Throughput | 0.75434 |
| GJA1 | Affinity Capture-MS | physical | High Throughput | 0.99174 |
| TMEM39A | Affinity Capture-MS | physical | High Throughput | 0.98233 |
| TMEM223 | Affinity Capture-MS | physical | High Throughput | 0.96551 |
| TMEM206 | Affinity Capture-MS | physical | High Throughput | 0.88227 |
| SUN1 | Affinity Capture-MS | physical | High Throughput | 0.91838 |
| SLC38A9 | Affinity Capture-MS | physical | High Throughput | 0.96765 |
| POMT1 | Affinity Capture-MS | physical | High Throughput | 0.99134 |
| SPG7 | Affinity Capture-MS | physical | High Throughput | 0.84027 |
| DAG1 | Affinity Capture-MS | physical | High Throughput | 0.94573 |
| ARL6IP5 | Affinity Capture-MS | physical | High Throughput | 0.84053 |
| LMF1 | Affinity Capture-MS | physical | High Throughput | 0.81525 |
| WRB | Affinity Capture-MS | physical | High Throughput | 0.8461 |
| TMX2 | Affinity Capture-MS | physical | High Throughput | 0.9351 |
| TUSC3 | Affinity Capture-MS | physical | High Throughput | 0.99996 |
| TMEM104 | Affinity Capture-MS | physical | High Throughput | 0.99611 |
| TMEM179B | Affinity Capture-MS | physical | High Throughput | 0.93065 |
| B4GALT1 | Affinity Capture-MS | physical | High Throughput | 0.96552 |
| FAM189B | Affinity Capture-MS | physical | High Throughput | 0.9412 |
| ALG10 | Affinity Capture-MS | physical | High Throughput | 0.94568 |
| TMEM231 | Affinity Capture-MS | physical | High Throughput | 0.99847 |
| CHST12 | Affinity Capture-MS | physical | High Throughput | 0.98213 |
| RHBDD1 | Affinity Capture-MS | physical | High Throughput | 0.99187 |
| LRRC8A | Affinity Capture-MS | physical | High Throughput | 0.99633 |
| ABHD16A | Affinity Capture-MS | physical | High Throughput | 0.99994 |
| GP1BB | Affinity Capture-MS | physical | High Throughput | 0.99347 |
| EDA | Affinity Capture-MS | physical | High Throughput | 0.87093 |
| SLC47A1 | Affinity Capture-MS | physical | High Throughput | 0.99571 |
| C10orf35 | Affinity Capture-MS | physical | High Throughput | 0.8971 |
| LRFN3 | Affinity Capture-MS | physical | High Throughput | 0.96675 |
| EMC8 | Affinity Capture-MS | physical | High Throughput | 0.88498 |
| LMF2 | Affinity Capture-MS | physical | High Throughput | 0.98646 |
| KIAA1715 | Affinity Capture-MS | physical | High Throughput | 0.83851 |
| RNF130 | Affinity Capture-MS | physical | High Throughput | 0.86551 |
| BRI3BP | Affinity Capture-MS | physical | High Throughput | 0.79698 |
| HTR3C | Affinity Capture-MS | physical | High Throughput | 0.87437 |
| ABHD16A | Affinity Capture-MS | physical | High Throughput | 0.99998 |
| UGT3A2 | Affinity Capture-MS | physical | High Throughput | 0.99988 |
| SEC11C | Affinity Capture-MS | physical | High Throughput | 0.99984 |
| HTR3C | Affinity Capture-MS | physical | High Throughput | 0.99983 |
| TPST2 | Affinity Capture-MS | physical | High Throughput | 0.99964 |
| TUSC3 | Affinity Capture-MS | physical | High Throughput | 0.9993 |
| TMEM186 | Affinity Capture-MS | physical | High Throughput | 0.99872 |
| TMTC3 | Affinity Capture-MS | physical | High Throughput | 0.99849 |
| SPPL2B | Affinity Capture-MS | physical | High Throughput | 0.99711 |
| TMEM104 | Affinity Capture-MS | physical | High Throughput | 0.99578 |
| METTL25 | Affinity Capture-MS | physical | High Throughput | 0.99537 |
| SLC38A9 | Affinity Capture-MS | physical | High Throughput | 0.99305 |
| FAM69A | Affinity Capture-MS | physical | High Throughput | 0.98794 |
| TMEM231 | Affinity Capture-MS | physical | High Throughput | 0.98728 |
| SLC47A1 | Affinity Capture-MS | physical | High Throughput | 0.98182 |
| WLS | Affinity Capture-MS | physical | High Throughput | 0.98088 |
| SLC38A10 | Affinity Capture-MS | physical | High Throughput | 0.96379 |
| LRRC8A | Affinity Capture-MS | physical | High Throughput | 0.95214 |
| GP1BB | Affinity Capture-MS | physical | High Throughput | 0.94525 |
| ALG9 | Affinity Capture-MS | physical | High Throughput | 0.93956 |
| EDA | Affinity Capture-MS | physical | High Throughput | 0.93542 |
| ERMP1 | Affinity Capture-MS | physical | High Throughput | 0.93368 |
| DAG1 | Affinity Capture-MS | physical | High Throughput | 0.929 |
| SLC35B2 | Affinity Capture-MS | physical | High Throughput | 0.92733 |
| GJA1 | Affinity Capture-MS | physical | High Throughput | 0.92686 |
| PIGM | Affinity Capture-MS | physical | High Throughput | 0.92333 |
| IMPAD1 | Affinity Capture-MS | physical | High Throughput | 0.91618 |
| PIGO | Affinity Capture-MS | physical | High Throughput | 0.90601 |
| LEMD3 | Affinity Capture-MS | physical | High Throughput | 0.89399 |
| POMT1 | Affinity Capture-MS | physical | High Throughput | 0.8921 |
| TMEM39A | Affinity Capture-MS | physical | High Throughput | 0.88403 |
| LRFN3 | Affinity Capture-MS | physical | High Throughput | 0.87404 |
| TMEM223 | Affinity Capture-MS | physical | High Throughput | 0.87191 |
| C10orf35 | Affinity Capture-MS | physical | High Throughput | 0.83693 |
| GRAMD1A | Affinity Capture-MS | physical | High Throughput | 0.80092 |
| ITPRIP | Affinity Capture-MS | physical | High Throughput | 0.79756 |
| TMED4 | Affinity Capture-MS | physical | High Throughput | 0.79099 |
| TMPPE | Affinity Capture-MS | physical | High Throughput | 0.78812 |
| CHST12 | Affinity Capture-MS | physical | High Throughput | 0.78267 |
| NETO2 | Affinity Capture-MS | physical | High Throughput | 0.77994 |
| GLT8D1 | Affinity Capture-MS | physical | High Throughput | 0.76826 |
